# Low-grade albuminuria and its relationship with cardiovascular disease risk in hypertensive and diabetic patients in primary health care

**DOI:** 10.1186/s12882-022-02884-7

**Published:** 2022-07-20

**Authors:** Ramon Augusto Ferreira de Souza, Eunice Ferreira da Silva, Deíse Moura de Oliveira, Renata Maria Colodette, Rosângela Minardi Mitre Cotta, Luciana Saraiva da Silva, Tiago Ricardo Moreira

**Affiliations:** 1 Departamento de Medicina e Enfermagem, Universidade Federal de Viçosa, Viçosa, MG Brazil; 2 Escola Nacional de Saúde Pública. Fundação Oswaldo Cruz, Rio de Janeiro, RJ Brazil; 3 Departamento de Nutrição e Saúde, Universidade Federal de Viçosa, Viçosa, MG Brazil; 4 Faculdade de Medicina. Universidade Federal de Uberlândia, Uberlândia, MG Brazil

**Keywords:** Cardiovascular diseases, Risk factors, Hypertension, Diabetes Mellitus, Albuminuria

## Abstract

**Objective:**

To evaluate the presence of LGA and the relationship with the 10-year risk of a cardiovascular event in hypertensive and diabetic patients in Primary Health Care.

**Study design:**

The study design used is cross-sectional.

**Methods:**

This study was based on the application of questionnaires, anthropometric measurements, and laboratory tests carried out from August 2017 to April 2018. Logistic regression was used to evaluate the odds ratio of the explanatory variables in relation to the highest tercile of LGA. The Framingham risk score was used to assess the 10-year risk of cardiovascular event. The comparison of this score with the LGA terciles was analyzed using ANOVA.

**Results:**

An increase in the 10-year risk of cardiovascular event score was observed with an increasing LGA tercile, and this pattern prevailed after adjusting for confounding variables.

**Conclusion:**

An association between LGA and the 10-year risk of cardiovascular event was observed in a representative sample of hypertensive and diabetic patients.

## Introduction

Moderately increased albuminuria is defined as the excretion of 30–300 mg albumin within 24 hours (or 20–200 mcg/min, or 30–300 mcg/mg creatinine) [1]. Recent studies indicate that low-grade albuminuria (LGA), when albuminuria levels are lower than 30 mg/g, is associated with cardiovascular disease and, as a result, LGA has been considered an important prognostic marker of cardiovascular and/or renal risk in patients with non-communicable chronic diseases (NCDs) such as patients with diabetes, hypertension, and in the general population. The association of LGA with cardiovascular events and kidney damage occurs continuously, so even in small amounts of urinary excretion of albumin, there is a risk of developing such conditions [2].

It is known that the flow of albumin and other macromolecules in the vessel walls causes inflammation and a build-up of lipids. Moreover, there is scientific evidence that associates the presence of LGA with insulin resistance and hypertension, thus contributing to the onset of micro and macrovascular diseases [3].

The literature points out that with a slight elevation in the UACR (> 5.8 mg/g), it is possible to identify a clinically relevant marker for the risk of ischemic heart disease after adjusting for atherosclerosis risk factors, such as the male gender, hypertension, dyslipidemia, smoking, old age, and obesity [4]. The *Framingham Heart Study*, on the other hand, has evidenced that low-grade urinary albumin excretion was associated with an increased risk of cardiovascular disease and mortality in non-hypertensive, non-diabetic individuals [5]. However, few studies have investigated the association of LGA with cardiovascular risk factors in individuals presenting hypertension and/or diabetes [6, 7].

This study is substantiated by contributing with epidemiological data on LGA in hypertensive and/or diabetic patients in Primary Health Care (PHC), which can assist healthcare providers and managers in the development of policies and measures that benefit the health of this population. Therefore, this study aimed to assess the prevalence of LGA and its relationship with cardiovascular disease risk in patients with hypertension and/or diabetes in PHC in the city of Viçosa, Minas Gerais, Brazil.

## Methods

The data used in the present study stems from the project entitled: “Prevention of diseases and illnesses in patients with arterial hypertension in the context of Primary Health Care: chronic kidney disease in focus” [8]. This is a cross-sectional study with a representative sample of hypertensive and/or diabetic individuals with no previous diagnosis of chronic kidney disease, receiving PHC in a city in the southeastern region of Brazil, carried out from August 2017 to April 2018. The project was approved by the Human Research Ethics Committee of the Universidade Federal de Viçosa (CEP/UFV), under legal opinion number 1203.173 (CAAE: 47356115.3.0000.5153). All ethical precepts of Resolution No. 466/2012 of the National Health Council were followed and the informed consent form was signed by all study participants.

The project participants were recruited from 16 of the 18 Basic Health Units of this city. The inclusion criteria for the participants were the following: to be aged over 18 years, to have been diagnosed with high blood pressure (HBP) and/or Diabetes (DM), to be assisted by the Family Health Strategy, and to agree to participate in the study after clarification. Participants who did not attend or could not come to the collection site on the scheduled day, pregnant women, individuals with severe clinical conditions requiring specialized care, and those with a history of alcohol and/or drug abuse were excluded.

A representative sample was collected from the city’s Basic Health Units. Conglomerate sampling was used in two stages, as follows: in the first stage, the health centers were sampled; in the second stage, patients under treatment in these centers were selected. The random selection of patients was carried out using the patient records of these health centers.

To calculate the project sample, the population of 6624 hypertensive and/or diabetic individuals registered and assisted by the PHC units in 2017 were considered, with 50% of estimated prevalence of the phenomenon, 5% margin of sampling error, 50% of conglomerate effect, 10% of refusals and/or losses, 20% to control for confounding factors, and 95% confidence level. The sample calculation was performed using the Statcalc program from Epi-Info® version 7.2, and the resulting sample size consisted of 719 individuals. A total of 841 individuals were recruited. In the present study, 91 individuals older than 75 years, 78 individuals with albuminuria ≥30 mg, and 35 individuals who did not participate in all stages of data collection were excluded, resulting in a final sample of 637 individuals for the present analysis (229 men and 408 women).

### Data Collection

Data collection was carried out through the application of questionnaires and the evaluation of anthropometric, clinical, and biochemical parameters. The questionnaire contained sociodemographic, clinical, and living habits variables and was based on previous studies [9, 10]. Systolic and diastolic blood pressures were measured with previously calibrated aneroid manometers and according to the procedures recommended by the Brazilian Guidelines on Arterial Hypertension [3].

The collection and analysis of biological materials (blood and urine) were performed in a commercial laboratory, which was licensed for this purpose, using commercial kits and reference standards of the laboratory’s own results. The participants were instructed on the procedures for collecting the first urine sample collected in the morning and on how to follow overnight fasting for the twelve hours preceding the blood collection. The biochemical tests performed were the following: fasting glucose (mg/dL), glycosylated hemoglobin (%), triglycerides (mg/dL), total cholesterol and fractions (mg/dL), phosphorus (mg/dL), calcium (mg/dL), serum albumin (mg/dL) and urinary creatinine (mg/dL) and albumin (mg/dL).

The measurement of albuminuria was performed based on the urine albumin-to-creatinine ratio in urine samples collected in the morning and analyzed by the nephelometric method. After excluding the individuals who presented a level of albuminuria greater than or equal to 30 mg/g, the remaining participants were divided into three groups according to the tercile of albuminuria (≤3.0, 4.0–7.0, ≥8.0). The highest tercile (≥8 mg/g) was adopted as the threshold of the albumin/creatinine ratio in urine, classifying these individuals as presenting LGA, without corrections for gender.

The *Framingham Risk Score* (FRS) was used to assess the risk of an individual presenting cardiovascular events in the next 10 years [3]. It is calculated based on the following variables: age, gender, total cholesterol, HDL-C, systolic blood pressure, and smoking [11]. Based on the FRS, individuals are categorized into three groups, namely: high-risk (> 20%), intermediate-risk (10–20%), and low-risk (< 10%) patients. An FRS ratio that is ≥20% is considered equivalent to coronary heart disease risk, and primary prevention is recommended for individuals in this category [11].

To characterize the population regarding the variables under study, a descriptive analysis was performed by estimating the absolute and relative frequencies, means, medians, standard deviations, and interquartile ranges. Pearson’s chi-square test was used to verify the associations between the categorical variables. The normality of continuous variables distribution was tested using the Shapiro Wilk test. According to the normality test results, either Student’s t-test (parametric) or the Mann Whitney test (nonparametric) was used.

To compare demographic, clinical characteristics and medication use between groups according to albuminuria terciles, ANOVA stratified by gender was performed. A logistic regression analysis was used to evaluate the odds ratio for the highest tercile of albuminuria and FRS having as covariates those that presented *p* < 0.200 in the previous analysis. The odds ratio and their respective 95% confidence intervals were used to assess the magnitude of the associations. The comparison of FRS with albuminuria terciles was analyzed using an ANOVA. The distribution of categorized FRS (low, medium, and high-risk) by albuminuria terciles were analyzed by the Chi-squared test. All tests were two-tailed, and *p*-values that were < 0.05 were considered to indicate statistical significance. All analyses were carried out using the *Statistical Package for the Social Sciences*, version 22; SPSS Inc. Chicago, USA.

## Results

### Demographic and clinical characteristics of the studied population

The demographic and clinical characteristics of the studied population are presented in Table 1. The mean age of individuals presenting HBP and/or DM evaluated was 61.2 years, and the mean BMI was 29.0 Kg/m^2^ (SD = ±5.6 kg/m^2^). Most were female, presenting with hypertension and metabolic syndrome. A 10-year risk of cardiovascular event greater than 20% was noted in 42.2% of the sample. Higher frequencies of smoking, obesity, and metabolic syndrome were found in women. While higher frequencies of alcohol use and 10-year cardiovascular event risk greater than 20% were found in men. Higher median values for FRS were obtained for males when compared to females. By further comparing the groups by gender, no great discrepancies were observed in the systolic arterial pressure values, and men presented higher mean diastolic arterial pressure values. Regarding the biochemical tests, women presented the highest rates of triglycerides and HDL. Regarding medication use, women had higher frequencies of use of statins, diuretics, adrenergic blockers and insulin.

**Table 1:** The frequency of demographic, clinical, and biochemical variables in patients presenting systemic high blood pressure (HBP) and/or *diabetes mellitus* (DM) without chronic kidney disease living in Viçosa - MG

VARIABLES	Total	Male	Female	***p***-value
	**n (%)**			
**Total**	637 (100)	229 (35.9)	408 (64.1)	–
**Smokers**	71 (11.1)	33 (46.5)	38 (53.5)	0.050*
**Alcohol users**	174 (27.7)	108 (62.1)	66 (37.9)	< 0.001*
**HBP**^**a**^	588 (92.3)	206 (35.0)	382 (65.0)	0.095*
**Obesity**	184 (28.9)	37 (20.1)	147 (79.9)	< 0.001*
**Diabetes**	292 (45.8)	109 (37.3)	183 (62.7)	0.505*
**Cardiovascular risk > 20%** ^**b**^	269 (42.2)	154 (57.2)	115 (42.8)	< 0.001*
**Metabolic syndrome**	404 (64.5)	112 (27.7)	292 (72.3)	< 0.001*
**Statins**	196 (30.8)	58 (29.6)	138 (70.4)	0.026*
**Lipid lowering medications**	7 (1.1)	3 (42.9)	4 (57.1)	0.706**
**Diuretics**	321 (50.4)	102 (31.8)	219 (68.2)	0.027*
**Adrenergic Blockers**	153 (24.0)	36 (23.5)	117 (76.5)	< 0.001*
**Direct-Acting Vasodilators**	13 (2.0)	4 (30.8)	9 (69.2)	0.779**
**Calcium Channel Blockers (CCBs)**	102 (16.0)	29 (28.4)	73 (71.6)	0.084*
**Angiotensin-Converting Enzyme Inhibitors (ACEIs)**	174 (27.3)	73 (42.0)	101 (58.0)	0.053*
**Angiotensin II AT1 Receptor Blockers (ARBs)**	234 (36.7)	74 (31.6)	160 (68.4)	0.083*
**Insulin**	57 (8.9)	28 (49.1)	29 (50.9)	0.030*
**Metformin**	186 (29.2)	67 (36.0)	119 (64.0)	0.981*
**Sulfonylureas**	65 (10.2)	20 (30.8)	45 (69.2)	0.358*
**SGLT2 inhibitor**	2 (0.3)	1 (50.0)	1 (50.0)	1.00**
**DPP-4 inhibitor**	3 (0.5)	1 (33.3)	2 (66.7)	1.00**
	**μ (Standard deviation)**			
**Mean age (years)**	61.2 ± 11.8	60.8 ± 12.3	61.4 ± 11.5	0.563**
**BMI**^**c**^ **(kg/m**^**2**^**)**	29.0 ± 5.6	27.3 ± 4.5	30.0 ± 6.0	< 0.001**
**SBP**^**d**^ **(mm/Hg)**	132.5 ± 18.2	133.0 ± 17.7	132.2 ± 18.4	0.595**
**DBP**^**e**^ **(mm/Hg)**	81.4 ± 10.5	82.6 ± 10.1	80.8 ± 10.7	0.035**
**Total cholesterol (mg/dL)**	191.4 ± 39.4	189.1 ± 39.2	192.7 ± 39.5	0.267**
**HDL-C**^**f**^ **(mg/dL)**	51.2 ± 13.0	48.2 ± 12.7	52.9 ± 12.9	< 0.001**
**GFR**^**g**^**(mL/min/1.73m**^**2**^**)**	84.5 ± 19.0	83.9 ± 20.1	84.8 ± 18.4	0.542**
	**Median (IR 25–75)**			
**Glycated hemoglobin (%)**	5.9 (5.6–6.8)	5.9 (5.6–6.7)	6.0 (5.6–6.9)	0.505***
**Glucose (mg/dL)**	97.0 (87.0–122.0)	97.0 (88.0–122.0)	96.5 (87.0–122.0)	0.133***
**Triglycerides (mg/dL)**	125.0 (95.0–170.0)	115.0 (86.0–171.0)	128.0 (101.5–169.5)	0.043***
**Urine albumin-to-creatinine ratio**	5.0 (3.0–8.0)	4.0 (3.0–9.0)	5.0 (3.0–8.0)	0.204***
**Framinghan score (points)**	16.9 (9.5–28.3)	27.8 (17.2–36.7)	12.7 (8.1–21.2)	< 0.001***

* Chi-squared test; ** T-test; *** Mann Whitney test; IR: interquartile range

^a^ high blood pressure; ^b^ 10-year risk of cardiovascular event greater than 20%; ^c ^body mass index; ^d^ systolic blood pressure; ^e^ diastolic blood pressure; ^f^ High-density lipoprotein (HDL) cholesterol; ^g ^glomerular filtration rate

### Demographic and clinical characteristics according to terciles of albuminuria

The results obtained for male and female subjects are presented in Tables 2 and 3. In mens, the glucose levels, glycated hemoglobin, and the cardiovascular risk score increased along with the increase in albuminuria terciles. A decrease in the frequency of diuretic use was observed with an increase in the albuminuria tertile. For women, an increase in age, glycated hemoglobin, use of sulfonylureas and cardiovascular risk score was observed from increasing albuminuria terciles. A decrease in total mean serum cholesterol levels was observed from increasing albuminuria terciles.

**Table 2:** The demographic and clinical characteristics according to the terciles of low-grade albuminuria, adjusted for the male population, Viçosa – MG

VARIABLES	Low-grade albuminuria terciles (unit)			***p***-value
	Tercile 1 (≤ 3.0)	Tercile 2 (4.0–7.0)	Tercile 3 (≥ 8.0)	
	**n (%)**			
**Smokers**	14 (42.4)	10 (30.3)	9 (27.3)	0.899*
**Alcohol users**	41 (38.0)	31 (28.7)	36 (33.3)	0.226*
**Obesity**	14 (37.8)	12 (32.4)	11 (29.7)	0.979*
**HBP**^**a**^	78 (37.9)	66 (33.0)	60 (29.1)	0.616*
**Diabetes**	37 (33.9)	38 (34.9)	34 (31.2)	0.340*
**Cardiovascular risk > 20%**^**b**^	54 (35.1)	51 (33.1)	49 (31.8)	0.156*
**Metabolic syndrome**	45 (40.2)	36 (32.1)	31 (27.7)	0.882*
**Statins**	18 (31.0)	19 (32.8)	21 (36.2)	0.235*
**Lipid lowering medications**	0 (0.0)	1 (33.3)	2 (66.7)	0.253*
**Diuretics**	38 (37.3)	42 (41.2)	22 (21.6)	0.028*
**Adrenergic Blockers**	10 (27.8)	17 (47.2)	9 (25.0)	0.119*
**Direct-Acting Vasodilators**	1 (25.0)	1 (25.0)	2 (50.0)	0.623*
**Calcium Channel Blockers (CCBs)**	8 (27.6)	13 (44.8)	8 (27.6)	0.276*
**Angiotensin-Converting Enzyme Inhibitors (ACEIs)**	27 (37.0)	24 (32.9)	22 (30.1)	0.899*
**Angiotensin II AT1 Receptor Blockers (ARBs)**	24 (32.4)	30 (40.5)	20 (27.0)	0.196*
**Insulin**	11 (39.3)	9 (32.1)	8 (28.6)	0.997*
**Metformin**	23 (34.3)	24 (35.8)	20 (29.9)	0.655*
**Sulfonylureas**	7 (35.0)	6 (30.0)	7 (35.0)	0.789*
**SGLT2 inhibitor**	0 (0.0)	1 (100.0)	0 (0,0)	0.357*
**DPP-4 inhibitor**	0 (0.0)	0 (0.0)	1 (100.0)	0.282*
	**μ (Standard deviation)**			
**Age (years)**	59.1 ± 11.7	61.2 ± 12.1	62.8 ± 13.0	0.170**
**BMI**^**c**^ **(kg/m**^**2**^**)**	27.3 ± 4.5	27.6 ± 4.8	26.9 ± 4.3	0.638**
**SBP**^**d**^**(mm/Hg)**	132.2 ± 17.3	132.0 ± 18.1	135.2 ± 17.8	0.485**
**DBP**^**e**^ **(mm/Hg)**	81.3 ± 10.5	83.1 ± 10.2	83.8 ± 9.3	0.257**
**Total cholesterol (mg/dL)**	187.0 ± 37.5	187.7 ± 38.4	193.7 ± 42.6	0.532**
**HDL**^**f**^ **(mg/dL)**	48.0 ± 12.6	49.0 ± 12.1	47.4 ± 13.8	0.755**
**GFR**^**g**^**(mL/min/1.73m**^**2**^**)**	83.0 ± 16.5	87.0 ± 20.0	81.5 ± 24.0	0.238**
**Framinghan score (points)**	26.7 ± 17.1	28.6 ± 15.7	33.6 ± 17.8	0.042**
	**median (IR 25–75)**			
**Glycated hemoglobin (%)**	5.7 (5.5–6.2)	6.0 (5.7–6.9)	6.0 (5.6–6.9)	0.005***
**Glucose (mg/dL)**	94.0 (86.0–114.0)	96.0 (90.0–141.0)	105.0 (90.0–135.0)	0.021***
**Triglycerides (mg/dL)**	113.0 (89.0–152.0)	112.0 (84.0–165.0)	137.0 (89.0–198.0)	0.213***

* Chi-squared test; ** ANOVA; *** Wilcoxon test; IR: interquartile range

^a^ high blood pressure; ^b^ 10-year risk of cardiovascular event greater than 20%; ^c^ body mass index; ^d^ systolic blood pressure; ^e^ diastolic blood pressure; ^f^ High-density lipoprotein (HDL) cholesterol; ^g ^glomerular filtration rate

**Table 3:** The demographic and clinical characteristics according to the terciles of low-grade albuminuria, adjusted for the female population, Viçosa – MG

VARIABLES	Low-grade albuminuria terciles (unit)			***p***-value
	Tercile 1 (≤ 3.0)	Tercile 2 (4.0–7.0)	Tercile 3 (≥ 8.0)	
	**n (%)**			
**Smokers**	11 (28.9)	14 (36.8)	13 (34.2)	0.679*
**Alcohol users**	23 (34.8)	27 (40.9)	16 (24.2)	0.692*
**Obesity**	46 (31.3)	61 (41.5)	40 (27.2)	0.543*
**HBP**^**a**^	129 (33.8)	145 (38.0)	108 (28.3)	0.942*
**Diabetes**	54 (29.5)	67 (36.6)	62 (33.9)	0.072*
**Cardiovascular risk > 20%**^**b**^	33 (28.7)	44 (38.3)	38 (33.0)	0.311*
**Metabolic syndrome**	96 (32.9)	108 (37.0)	88 (30.1)	0.568*
**Statins**	50 (36.2)	48 (34.8)	40 (29.0)	0.599
**Lipid lowering medications**	1 (20.0)	2 (50.0)	1 (25.0)	0.879
**Diuretics**	80 (36.5)	78 (35.6)	61 (27.9)	0.371
**Adrenergic Blockers**	39 (33.3)	41 (35.00)	37 (31.6)	0.617
**Direct-Acting Vasodilators**	3 (33.3)	2 (22.2)	4 (44.4)	0.489
**Calcium Channel Blockers (CCBs)**	26 (35.6)	28 (38.4)	19 (26.0)	0.863
**Angiotensin-Converting Enzyme Inhibitors (ACEIs)**	33 (32.7)	34 (33.7)	34 (33.7)	0.370
**Angiotensin II AT1 Receptor Blockers (ARBs)**	55 (34.4)	66 (41.2)	39 (24.4)	0.315
**Insulin**	11 (37.9)	8 (27.6)	10 (34.5)	0.480
**Metformin**	35 (29.4)	46 (38.7)	38 (31.9)	0.447
**Sulfonylureas**	11 (24.4)	14 (31.1)	20 (44.4)	0.040
**SGLT2 inhibitor**	0 (0.0)	1 (100.0)	0 (0.0)	0.441
**DPP-4 inhibitor**	2 (100.0)	0 (0.0)	0 (0.0)	0.137
	**μ (Standard deviation)**			
**Age (years)**	59.1 ± 11.6	61.3 ± 11.0	64.2 ± 11.6	0.002**
**BMI**^**c**^ **(kg/m**^**2**^**)**	30.2 ± 5.7	30.6 ± 6.6	29.1 ± 5.3	0.139**
**SBP**^**d**^**(mm/Hg)**	130.1 ± 16.6	131.7 ± 16.8	135.3 ± 22.0	0.074**
**DBP**^**e**^ **(mm/Hg)**	80.9 ± 10.8	80.2 ± 10.5	81.3 ± 10.9	0.674**
**Total cholesterol (mg/dL)**	196.5 ± 38.7	195.7 ± 38.6	184.3 ± 40.7	0.025**
**HDL**^**f**^ **(mg/dL)**	54.3 ± 13.0	51.8 ± 12.2	52.6 ± 13.7	0.236**
**GFR**^**g**^**(mL/min/1.73m**^**2**^**)**	82.7 ± 17.4	87.0 ± 18.1	84.5 ± 19.9	0.141**
**Framinghan score (points)**	14.2 ± 10.8	16.2 ± 11.6	18.1 ± 12.6	0.029**
	**median (min – max)**			
**Glycated hemoglobin (%)**	5.9 (5.6–6.0)	5.9 (5.5–6.8)	6.2 (5.8–7.3)	0.006***
**Glucose (mg/dL)**	95.0 (87.0–118.0)	96.0 (87.0–120.0)	101.5 (87.5–141.0)	0.216***
**Triglycerides (mg/dL)**	120.0 (93.0–163.0)	136.0 (104.0–177.0)	126.5 (102.5–165.0)	0.051***

* Chi-squared test; ** ANOVA; *** Wilcoxon test; IR: interquartile range

^a^ high blood pressure; ^b^ 10-year risk of cardiovascular event greater than 20%; ^c^ body mass index; ^d^ systolic blood pressure; ^e^ diastolic blood pressure; ^f^ High-density lipoprotein (HDL) cholesterol; ^g ^glomerular filtration rate

**Fig. 1:**
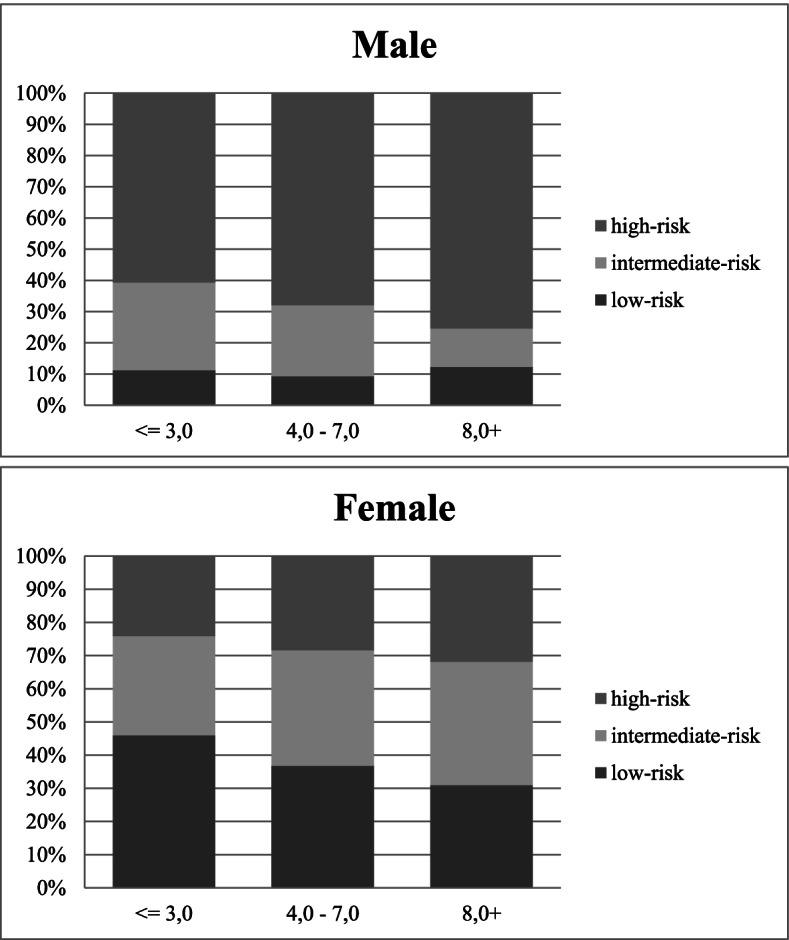
Distribution of the 10-year Framingham risk by gender and tercile of low-grade albuminuria. The 10-year risk of coronary heart disease according to the Framingham score: > 20% = high-risk; 10–20% = intermediate-risk, and < 10% = low-risk instances

The distribution of 10-year CVD risk by gender according to FRS in relation to each tercile of LGA is presented in Fig. 1. For males, an increase in the percentage of high risk of cardiovascular event was noted according to the increase in the albuminuria tercile. For women, a slight increase in the frequency of moderate and high risk is noted as the albuminuria tercile increases.

Table 4 presents the final logistic regression model for evaluating the factors associated with the highest tercile of albuminuria. The logistic regression analysis found that total cholesterol, age, and triglycerides were associated with the highest tercile of albuminuria. The increase of one year of age and 1 mg/dl of triglycerides caused albuminuria to increase by 0.03 and 0.002 mg/g, respectively. The increase of 1 mg/dl of total cholesterol reduced albuminuria by 0.005 mg/g.

**Table 4:** The final logistic regression model for the highest tercile of albuminuria*

	Odds ratio (IC:95%)	***p***-value
**Total cholesterol**	0.995 (0.990–1.000)	0.044
**Age**	1.029 (1.012–1.046)	0.001
**Triglycerides**	1.002 (1.000–1.005)	0.044
**Body mass index**	0.965 (0.930–1.001)	0.054
**Glycated hemoglobin**	1.112 (0.986–1.253)	0.083
**Use of Direct-Acting Vasodilators**	2.924 (0.918–9.313)	0.069

* Urine albumin-to-creatinine ratio (UACR)

The mean values of the 10-year risk of cardiovascular event score increased as the tercile of albuminuria increased (unadjusted ANOVA). This pattern prevailed despite adjustments for gender, diabetes, hypertension, glomerular filtration rate < 60 mL/min/1.73m^2^, and metabolic syndrome (Table 5).

**Table 5:** Adjustment of the 10-year CVD risk score by tercile of low-grade albuminuria

	Tercile 1 (0–3.0)	Tercile 2 (4.0–7.0)	Tercile 3 (8.0–29.9)	***p***-value
**Non-Adjusted**	19.117 ± 1.006	20.205 ± 0.998	23.651 ± 1.124	0.009
**Adjusted for gender**	18.659 ± 0.913	20.651 ± 0.905	23.656 ± 1.019	0.001
**Adjusted for age**	20.554 ± 0.857	20.139 ± 0.845	21.942 ± 0.959	0.349
**Adjusted for gender and age**	20.107 ± 0.737	20.594 ± 0.727	21.921 ± 0.824	0.249
**Adjusted for diabetes**	19.606 ± 0.967	20.222 ± 0.956	23.020 ± 1.081	0.048
**Adjusted for HBP**^**a**^	19.164 ± 1.001	20.186 ± 0.992	23.618 ± 1.118	0.009
**Adjusted for GFR**^**b**^ **< 60**	19.315 ± 0.999	20.222 ± 0.989	23.383 ± 1.117	0.020
**Adjusted for MS**^**c**^	19.218 ± 1.014	20.239 ± 1.005	23.689 ± 1.124	0.010
**Adjusted for gender, age, diabetes, MS, GFR < 60, DBP**^**d**^**, HDL**^**e**^**, smokers, and obesity**	21.181 ± 0.616	20.632 ± 0.604	20.788 ± 0.682	0.810

^a^ high blood pressure; ^b^ glomerular filtration rate provided in mL/min/1.73m^2^; ^c^ metabolic syndrome; ^d^ diastolic blood pressure; ^e^ High-density lipoprotein

## Discussion

Microalbuminuria, a urine albumin excretion ranging from 30 to 300 mg/day, is described as a marker of endothelial dysfunction [6, 12–14], and glomerular hyperfiltration [6], and is correlated with the structural and functional integrity of the vasculature [13]. Subtle changes in albumin excretion reflect generalized vascular processes and highlight the complex association between chronic kidney disease and cardiovascular disease [13]. Microalbuminuria is a known risk factor for metabolic syndrome [15], cardiovascular disease [16], and peripheral arterial disease [17]. It is associated with cardiovascular morbidity and mortality in patients presenting with hypertension [7], in individuals presenting with or without diabetes [18], or with end-stage renal disease in individuals with an adverse cardiovascular risk profile [12, 18]. It is common in the general population, especially in patients with DM or HBP [12].

However, studies indicate that lower levels of UACR, previously considered within the normal range, associate LGA with cardiovascular morbidity and mortality in the general population [18]. Urine albumin excretion predicts blood pressure progression in non-diabetic and non-hypertensive individuals incrementally in relation to established risk factors and at levels well below the conventional threshold for microalbuminuria [6]. In *The Framingham Heart Study*, which was carried out with middle-aged, non-hypertensive, and non-diabetic individuals, urine excretion of LGA predicted the development of cardiovascular diseases [5, 18], challenging the indication of the limit for albumin excretion considered normal^5^ and suggesting that this level, in the general population, is much lower [13].

The results of this study demonstrate a significant association between LGA and 10-year CVD risk in a sample of hypertensive and/or diabetic patients assisted by PHC. Several other studies associate LGA with increased risk of all-cause cardiovascular disease [12, 13, 18–21], including apparently healthy individuals presenting no DM or HBP [12]. A study carried out with a nationally representative sample of 9736 adult Koreans associated albuminuria within the normal range with metabolic syndrome [18]. A study carried out with 1341 middle-aged and elderly Chinese adults, who were normotensive and euglycemic, presenting normal UACR ratios, suggested the existence of a contribution of LGA to the risk of atherosclerosis [16]. Another study carried out with 760 Chinese participants aged from 29 to 76 years, presenting type 2 DM and normoalbuminuria, has demonstrated a significant association between LGA and increased thickness of the carotid intima-media layer [20]. UACR was associated with subclinical left ventricular diastolic dysfunction and left ventricular remodeling in patients presenting with and without type 2 DM [19].

There is evidence suggesting that LGA, below the microalbuminuria threshold, is associated with an increased prevalence of metabolic syndrome and its components [15], systemic vascular dysfunction, and cardiovascular mortality [22]. LGA is prevalent in patients presenting hypertension and is associated with an unfavorable outcome [22], it may identify individuals who are more likely to develop HBP [6], it may also be used to detect early cardiovascular disease in patients presenting type 2 DM [20], it may contribute to the risk of carotid atherosclerosis [23], and it may also be used to independently predict the incidence of cardiovascular disease and mortality in apparently healthy individuals with optimal blood pressure, without diabetes, and in the general population [24]. LGA has also been associated with left ventricular hypertrophy and left ventricular diastolic dysfunction in hypertensive patients [7], and is a potentially important risk factor for ischemic stroke, especially for the lacunar subtype [14].

LGA may also be an important marker of subclinical cardiovascular damage [19] and be used as an early marker for the detection of atherosclerosis in patients presenting type 2 DM [23], for the detection of peripheral arterial disease in diabetic patients [17], as well as being useful as an early biomarker for cardiovascular risk and mortality [18, 23, 24]. LGA also seems to be a more important determinant in the early stages of chronic kidney disease than glomerular filtration rate [13].

Finally, there may be some clinical relevance for albuminuria measurement tests, which are simple [25], widely available commercially [14, 25], inexpensive, and reliable [14]. These are interactive tests, which are used in combination as a preliminary instrument in the diagnosis and prognosis of chronic kidney disease [25], and also offer an evaluation of the risk of endothelial dysfunction, in order to provide screening for diseases, comparable to the role of blood pressure and lipids [14], in addition to predicting adverse outcomes, including mortality [25]. Therefore, these tests should be regularly performed in the elderly, in patients presenting DM, HBP, and cardiovascular diseases [25].

In addition to the results considered and consistent with data already described in the literature, other factors that make this study relevant are the representative sample and the pioneering approach in the city and region. However, the sample size makes the results of the present study more exploratory in nature and, ideally, should constitute a springboard for a larger and more representative study. Other limitations of the study were related to the failure to use the 24-hour urine collection technique when obtaining these samples and also to the lack of comparison of data in individuals without high blood pressure and diabetes.

Therefore, the results of the present study suggest that the screening for LGA can be a potential ally in preventive work in the Family Health Strategies and it requires a multidisciplinary approach, making its use necessary as an instrument to stratify hypertensive and diabetic patients assisted in Primary Health Care units. Moreover, it highlights the need for the development of effective public policies and the development of protocols that can be effective and put into practice by frontline healthcare providers working in the Brazilian Unified Health System.

It is concluded that LGA was prevalent and significantly associated with cardiovascular risk in patients diagnosed with HBP and/or DM, proving to be an important indicator to estimate the 10-year risk of cardiovascular event, with potential for routine use in PHC.

## Data Availability

The datasets used and/or analyzed during the current study are available from the corresponding author upon reasonable request.
